# A cellular automaton model for spheroid response to radiation and hyperthermia treatments

**DOI:** 10.1038/s41598-019-54117-x

**Published:** 2019-11-27

**Authors:** Sarah C. Brüningk, Peter Ziegenhein, Ian Rivens, Uwe Oelfke, Gail ter Haar

**Affiliations:** Joint Department of Physics at The Institute of Cancer Research and the Royal Marsden NHS Foundation Trust, London, UK

**Keywords:** Computational models, Multicellular systems, Cancer models

## Abstract

Thermo-radiosensitisation is a promising approach for treatment of radio-resistant tumours such as those containing hypoxic subregions. Response prediction and treatment planning should account for tumour response heterogeneity, e.g. due to microenvironmental factors, and quantification of the biological effects induced. 3D tumour spheroids provide a physiological *in vitro* model of tumour response and a systems oncology framework for simulating spheroid response to radiation and hyperthermia is presented. Using a cellular automaton model, 3D oxygen diffusion, delivery of radiation and/or hyperthermia were simulated for many (${\bf{1}}{{\bf{0}}}^{{\bf{4}}}{\boldsymbol{-}}{\bf{1}}{{\bf{0}}}^{{\bf{7}}}$) individual cells forming a spheroid. The iterative oxygen diffusion model was compared to an analytical oxygenation model and simulations were calibrated and validated against experimental data for irradiated (0–10 Gy) and/or heated (0–240 *CEM*_43_) HCT116 spheroids. Despite comparable clonogenic survival, spheroid growth differed significantly following radiation or hyperthermia. This dynamic response was described well by the simulation (${{\bf{R}}}^{{\bf{2}}}$ > 0.85). Heat-induced cell death was implemented as a fast, proliferation-independent process, allowing reoxygenation and repopulation, whereas radiation was modelled as proliferation-dependent mitotic catastrophe. This framework stands out both through its experimental validation and its novel ability to predict spheroid response to multimodality treatment. It provides a good description of response where biological dose-weighting based on clonogenic survival alone was insufficient.

## Introduction

The combination of radiotherapy (RT) with hyperthermia (HT, prolonged elevation of temperature above the physiological range) provides a promising treatment for radiation-resistant tumours by invoking a mechanism for localized heat-induced radiosensitization, in cases for which ionising dose escalation is limited due to normal tissue toxicity. The biological effects induced by RT and HT act synergistically^1–5^, but are strongly dependent on the heating temperature, duration and sequence of the combined treatment. Quantification of the biological effects produced is essential for treatment dose prescription and response prediction. Biological response quantification based on clonogenic cell survival data has been proposed^5–8^ with dose weighted by a relative biological effectiveness using heating time and temperature dependent parameters to calculate the biologically equivalent dose (BEQD) in units of RT only treatments. This type of biological effect calculation does not, however, account for the dynamics of tumour growth delay or shrinkage following treatment which is affected by the treatment modality due to differences in the cell death mechanisms induced^9,10^. In addition, it has been shown that cells cultured in 2D, as used for clonogenic assays, may respond differently to treatment than more physiologically relevant 3D tumour spheroid cultures^11–13^ raising concerns about the applicability of biological dose weighting purely based on clonogenic cell survival assays. Tumour spheroids are avascular 3D aggregates of cells which mimic the physiological microenvironment of tumours *in vivo* more closely than 2D cultures by providing gradients of oxygen, nutrients and waste products. Cells are in direct (3D) contact with each other, thus stimulating further inter-cellular communication and allowing interaction between cells and their extra-cellular matrix. Tumour spheroids are therefore considered to provide a simple *in vitro* tumour model that allows study of treatment-induced growth dynamics of the cell population as a whole, and may be a better *in vitro* model for quantifying and predicting *in vivo* tumour growth than 2D cultures^14–16^. Recent studies have investigated the dynamics of cell cycle progression and cell death within irradiated spheroids^9,10^ and suggested a proliferation dependence of radiation-induced cell death as a consequence of irreparable DNA damage and mitotic catastrophe^17,18^. Although investigations of spheroid response to HT or combined RTHT treatments have been performed^19–24^, these mainly focused on an analysis of spheroid growth delay, cell viability and dissociated spheroids clonogenic survival. An increased heat resistance of cells treated in spheroids has been suggested. Little information exists, on the dynamic process of heat-induced cell death and to what extent differences in mircoenvironmental factors and cell cycle distribution contribute to the observed increase in heat resistance. We suggest that within heated spheroids cells undergo a rapid, and proliferation-independent cell death. This agrees with the observation that several cellular components, including functional and structural proteins, are affected by this treatment^25,26^. Current BEQD models cannot account for these differences in the cell death mechanisms leading to microenvironmental changes and therefore are unable to explain the observed differences in spheroid growth following treatment with RT or HT at isoeffective (thermal) dose levels. Agent-based systems oncology simulation, such as cellular automaton models^27–29^, provide the mathematical tools required to meet this objective. Here, cells are modelled as individual voxels on a (3D) computational grid with specific growth and treatment response properties. Dynamic changes in microenvironmental factors (e.g. oxygenation) are also accounted for. We present a 3D cellular automaton (CA) model for simulating simultaneous RT and HT treatments, and compare it to experimental data obtained using spheroids formed from the human colorectal carcinoma cell line HCT116. The aim of this study is to demonstrate the importance of considering differences in the cell death mechanisms induced after RT or HT treatments and how these impact the dynamic growth response of the spheroid as a first step towards more sophisticated simulations and BEQD estimations for tumours *in vivo*. Where possible, a deliberately simple implementation was chosen in order to minimize the number of model parameters.

## Methods

### Experimental data

Simulations were compared to experimental data obtained for the colorectal carcinoma cell line HCT116 that will be published separately and describe in detail spheroid initiation, assessment of their growth, live cell imaging and preparation of histological sections from spheroids, as well as delivery of radiation and hyperthermia treatments for spheroid and clonogenic survival analysis. Briefly, cells were grown in Dulbecco’s modified Eagle’s medium (Gibco Life Technologies Ltd. Paisley, UK) supplemented with 10% fetal bovine serum (PAN Biotech GmbH, Aidenbach, Germany) under standard mammalian cell culture conditions (37 ${}^{\circ }C$, 5% CO${}_{2}$ in a humidified atmosphere). Spheroids were prepared by seeding 300 HCT116 cells in exponential growth phase, in U-shaped, ultra low attachment plates (ULA plates, #7007, Corning B.V. Life Sciences, Amsterdam, NL), and were incubated for four days prior to treatment. This allowed the cells in each well to form a single dense 3D aggregate, and gradients of nutrients and oxygen to be established. Spheroids were irradiated in a small animal radiation research platform (220 kVp, 13 mA, 0.14 mm Cu filtering, X-Strahl, Camberley, UK) and/or heated in a Biorad Tetrad2 DNA Engine PCR thermal cycler (Bio-Rad Laboratories Inc., Berkeley, USA) at 47 ${}^{\circ }C$ for various lengths of times, before being transferred in complete growth medium supplemented with 1% penicillin, streptomycin and amphotericin B (all Sigma Aldrich Ltd., Dorset, UK) and 5 $\mu g/ml$ propidium iodine (PI, Sigma Aldrich - which stains dead cells) to fresh ULA plates. Cultures were maintained for three weeks after treatment with the medium being renewed every three days. Spheroid diameter (growth) was assessed every other day using an automated Celigo^TM^ imaging cytometer (Nexcelom Biosciences LLC, Lawrence, USA). An implementation of a deep learning neural network was used for automated spheroid segmentation.

### Computational model

A previously published cellular automaton framework^30^ was extended to describe spheroid growth on a 3D lattice. A typical grid size was 200 $\times$ 200 $\times$ 200 cubic voxels, modelled with 0.012 mm edge length, corresponding to the average size of HCT116 cells obtained previously for 2D simulations^30^. Cells are initialized as forming a dense sphere on the grid, with each cell occupying one voxel. Cells progress through a predefined cycle (G1, S, G2, M-phases) according to an individualized timer and starting from a random cycle stage as described previously^30^. During cell division, next neighbours were searched alternately in the 3D Moore and vonNeumann neighbourhoods up to second order. Here, more central locations are favoured to ensure a compact, spherical structure. If no free space was available, cells entered a reversible quiescent stage (G0) until neighbouring spaces are vacated. In the absence of treatment, cells were simulated as having an infinite lifespan and unlimited division potential. However, oxygenation levels and the presence of free next neighbours influenced cell cycle progression and cell survival (death after prolonged hypoxia) as described in the following section. No cell migration within the spheroid was considered.

#### Oxygenation and untreated spheroid growth

The partial oxygen pressure ${p}_{O2}({\bf{x}},t)$ at 3D location ${\bf{x}}$ and time $t$ were modelled by solving the oxygen reaction diffusion equation iteratively:

1 $$\frac{{\rm{\partial }}{p}_{O2}({\bf{x}},t)}{{\rm{\partial }}t}={D}_{{O}_{2}}{\rm{\Delta }}{p}_{O2}({\bf{x}},t)-{\rm{\Phi }}({\bf{x}})$$

Here, ${D}_{{O}_{2}}$ is the oxygen diffusion coefficient in the medium, and ${\rm{\Phi }}({\bf{x}})$ denotes the cellular oxygen consumption rate where voxel ${\bf{x}}$ is occupied (${\rm{\Phi }}({\bf{x}})=0$ for unoccupied voxels). Boundary conditions are set to full oxygenation outside the spheroid volume corresponding to a partial oxygen pressure of 100 mmHg as previously reported by Grimes *et al*. for spheroid cultures within a CO${}_{2}$ incubator^31,32^. Since the time scale for oxygen diffusion (seconds) is far less than that of cell cycle progression (hours), the oxygen distribution is updated within each cell cycle iteration until an equilibrium stage is reached. Equilibrium was defined as a maximum change in oxygen partial pressure of $\le$0.01% at any location.

It is known that cells die if kept under hypoxic conditions for prolonged periods of time (chronic hypoxia)^33^. Lack of other nutrients, accumulation of cellular debris, and mechanical pressure are known to contribute to cell cycle arrest and cell death especially at the core of a spheroid^34–37^. For simplicity, only hypoxia-induced cell death was modelled in this framework, but the parameters may be regarded as accounting for several microenvironmental factors summarized under the term “hypoxia”.

Cells were labelled as hypoxic once the partial oxygen pressure fell below 11 mmHg^38^. These hypoxic cells died with probability ${p}_{hypoxiaDeath}$ at each simulation time step if they did not return to normoxic conditions, thus forming a necrotic core. Although the underlying mechanism is still subject to current research, it is agreed that the formation of a necrotic core results in spheroid growth plateauing over time. To account for this experimental observation in the simulation, necrotic cells were removed from the computational grid with probability ${p}_{clearNecrotic}$ at each time step, whereas a constant growth of the proliferating spheroid layer was assumed. All cells were continuously shuffled inwards in order to maintain a dense spherical structure. By choosing small values for ${p}_{hypoxiaDeath}$ and ${p}_{clearNecrotic}$ it was not necessary to define additional parameters to control the minimum time under hypoxia before cell death, or a minimum time after cell death before clearance. This implementation clearly does not reflect the actual biological processes, instead it provides a simplification that mimics the spheroid growth plateau while being restricted to a fixed-sized lattice.

#### Radiation response

Radiation-induced cell death in 3D spheroids was modelled as a proliferation dependent process. Cells were randomly divided into a dying, and a surviving population according to the clonogenic surviving fraction ${S}_{RT}$ that was calculated using the linear quadratic model^39^ with cell line specific parameters $\alpha$ and $\beta$ as described previously^7^. Weighting factors for cell cycle stage, $\gamma$, and oxygenation status, ${d}_{OER}$, were applied upon calculation of this surviving fraction.

2 $${S}_{RT}={e}^{-\gamma \cdot \left(\alpha \cdot {d}_{OER}+\beta \cdot {d}_{OER}^{2}\right)}$$

Here, the decreased radiosensitivity of hypoxic cells relative to normoxic ones was accounted for by weighting the radiation dose, $d$, by the oxygen enhancement ratio, $OER$, to obtain the apparent delivered dose, ${d}_{OER}$.

3 $${d}_{OER}=\frac{d}{OER({p}_{{O}_{2}})}\quad {\rm{with}}\quad {\rm{OER}}=\left\{\begin{array}{ll}1\quad {\rm{if}} & p{O}_{2} > 11\,mmHg\\ 3-\frac{2\cdot p{O}_{2}}{11\,mmHg}\quad {\rm{if}} & p{O}_{2}\le 11\,mmHg\end{array}\right.$$

As the ioseffective ratio of doses under normoxic and hypoxic condion, the oxygen enhancement ratio (OER)^40^ depends on the partial oxygen pressure $p{O}_{2}$. However, the specific cell line, and linear energy transfer of the radiation also contribute to the OER. Typical experimentally determined values for the OER are in the range 1–3^32^. This motivated the choice of low and high oxygenation level limits of the step function described in equation 3 using a linear interpolation for hypoxic oxygen levels.

**Figure 1:**
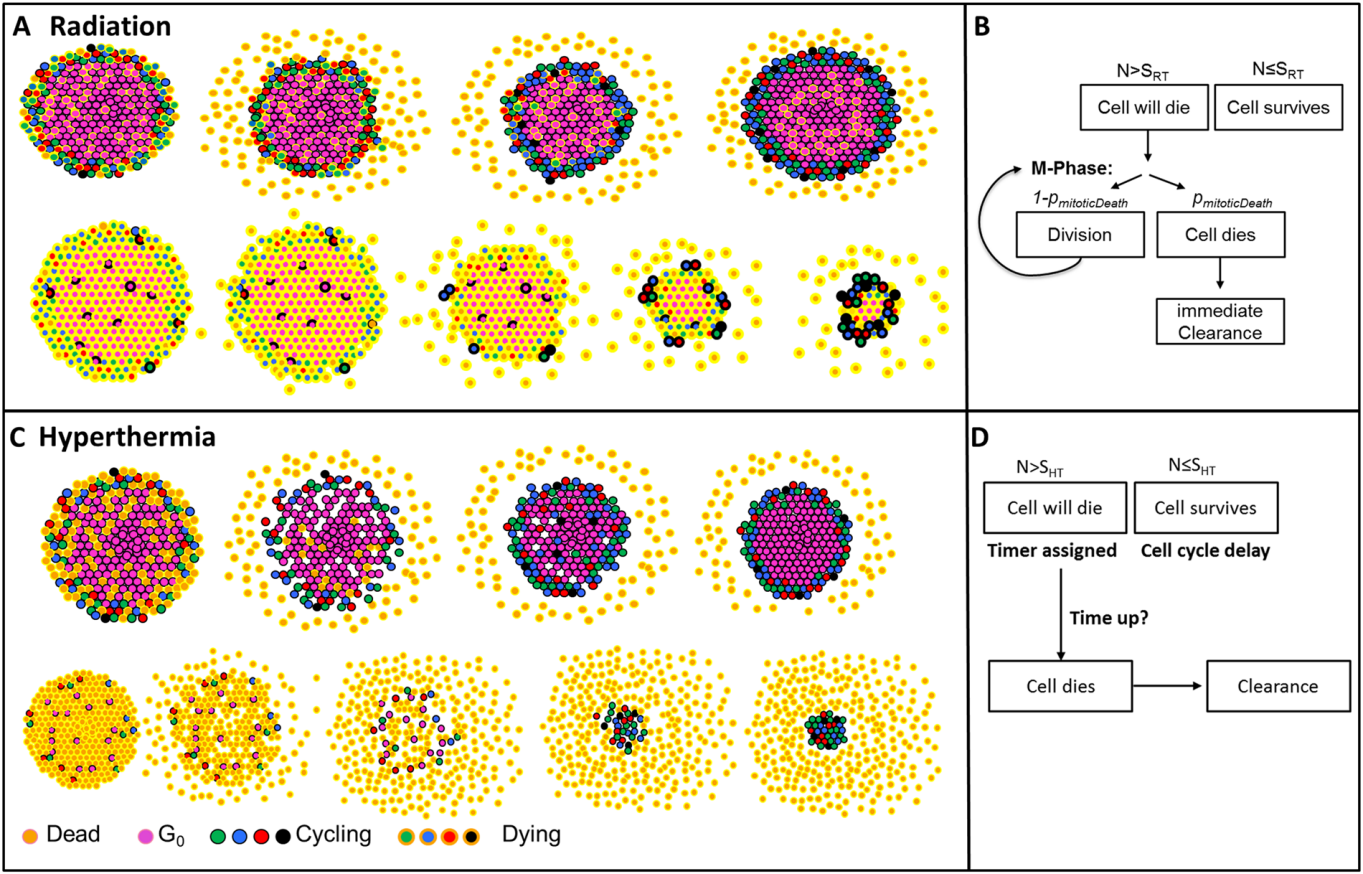
(**A**,**C**) Schematic diagrams showing the dynamics of cell death and cell detachment implemented for simulation of RT (**A**) or HT (**C**) treatments in a spheroid. Images are shown in chronological order from left to right, differences in cell cycle stage are indicated by colour for dying (yellow contour) and surviving (cycling, black contour) cells. (**B**,**D**) implemented probability driven response after RT and HT exposure. $N$ is a random number between 0 and 1 drawn for each cell, S is the surviving fraction calculated using equations 2 and 4. Following irradiation, a proportion $1-{S}_{RT}$ of cells is labelled as dying, but cell death is delayed until the particular cell attempts division. This implies that cell death is restricted to the proliferating zone of the spheroids and quiescent cells need to re-enter the cell cycle before being affected by radiation induced cell death. The response of a spheroid with a large fraction of surviving cells (top row in **A**) and a high dose irradiated spheroid (bottom row in **A**) is shown. Due to the reproduction of the surviving cells, dying quiescent cells may remain trapped at the centre of the spheroid. After HT, $1-{S}_{HT}$ cells are labelled as dying and are assigned a random delay to cell death independent of their location within the proliferating or quiescent zone of the spheroid. Once a cell dies (i.e. delay has ended), it is immediately removed from the computational grid resulting in a transient decrease in cell density, allowing previously quiescent cells (pink) to re-enter the cell cycle. The response of a spheroid with a large fraction of surviving cells (top row in **C**) and after treatment with a large thermal dose (bottom row in **C**) is shown.

Figure 1A,B shows a schematic diagram and the probability driven response cascade used to mimic radiation-induced cell death. Cells randomly selected to survive the radiation treatment (with probability ${S}_{RT}$) continued to proliferate, no cell cycle delay was included for surviving cells. “Dying cells” underwent mitotic cell death with probability ${p}_{mitoticDeath}$ each time they attempted division at the end of M-phase; with probability $1-{p}_{mitoticDeath}$ that an irradiated cell successfully divided into two daughter cells, both labelled as dying.

Experimentally, irradiated spheroids showed no signs of radiation-induced growth inhibition within the first days after exposure. This was accounted for in the simulation as a two stage process using two parameter values for ${p}_{mitoticDeath}$: within the initial days (parameter ${t}_{delayRT}$) ${p}_{mitoticDeath < 0.5}$, i.e. net growth, and ${p}_{mitoticDeath > 0.5}$, for the remaining time, resulting in spheroid shrinkage.

The implementation presented allows gradual shrinkage of irradiated spheroids from the outside inwards. Quiescent cells were sequentially reactivated and underwent mitotic death once attempting division. This resulted in a continuous shrinkage of a lethally irradiated spheroid (i.e no surviving cells), while maintaining a compact spherical structure. If the proportion of surviving cells was sufficiently large, spheroid regrowth took place before the inner-most quiescent cells re-enter the cell cycle. These dying quiescent cells remained within the spheroid core, encapsulated by layers of proliferating, surviving cells. Figure 1A illustrates the dynamic cell death after the irradiation described above for a high (very few surviving cells) and low (few dying cells) dose.

#### Hyperthermia treatments

For simulation, heated cells were separated into surviving and dying populations according to the clonogenic surviving fraction, ${S}_{HT}$, directly after heat treatment. Since the thermal-resistance observed in HCT116 spheroids was enhanced compared to their clonogenic survival in 2D monolayer cultures, adjustments were made to the clonogenic surviving fraction calculated using the AlphaR model^7^. It was assumed here that the 3D surviving fraction in response to heat treatment, ${S}_{HT}({t}_{43})$, followed a simple step-wise function of thermal dose, ${t}_{43}$, the 2D culture AlphaR model parameters ${\beta }_{HT}$ and ${\alpha }_{0}$, and the plateau surviving fraction ${S}_{HT,plateau}$:

4 $${S}_{HT}({t}_{43})=\left\{\begin{array}{ll}{S}_{HT}({t}_{43})\quad {\rm{asi\; neq.\; (5)}}\quad  & {\rm{if}}\quad {t}_{43}\le 160\,{CEM}_{43}\\ {S}_{HT,plateau} & {\rm{if}}\quad {t}_{43} > 160\,{CEM}_{43}\end{array}\right.$$

5 $$-log({S}_{HT}({t}_{43}))=\{\begin{array}{cc}{\gamma }_{HT}({\beta }_{HT}{t}_{43}^{2}) & {\rm{i}}{\rm{f}}\,{t}_{43}\le {D}_{T}=\frac{{\alpha }_{0}}{2{\beta }_{HT}}\\ {\gamma }_{HT}({\alpha }_{0}{t}_{43}-\frac{{\alpha }_{0}^{2}}{4{\beta }_{HT}})\, & {\rm{i}}{\rm{f}}\,{t}_{43} > {D}_{T}\end{array}$$

No adjustments were made to allow for differences in cell death as a function of the cell’s oxygenation level as previously suggested^41,42^. Although other parameters, such as a more acidic pH in the spheroid core, would enhance heat-induced killing, these were not considered in this simplified implementation. Cell cycle stage dependent heat sensitivity variation was considered, in particular differences between cycling and quiescent (G0) cells. This was achieved by using a weighting factor (${\gamma }_{HT}$) as described above for the modelling of radiation response. A constant ratio of 1.5 (as used in previous 2D simulations^30^) between G1, S, G2-phase cells was used. G0 cells were assumed to be more resistant by a factor of three relative to the average surviving fraction of the cycling cells, motivated by^43,p.19^.

Heat-induced cell death dynamics were implemented as occurring after a delay time (sampled from a normal distribution, mean value ${t}_{delayHTtodeath}$), followed by immediate clearance of dead cells. Surviving cells were simulated as continuing their cell cycle progression after a randomly assigned cycle arrest (${t}_{cellcycledelay}$), sampled from a uniform distribution. Free parameters for the HT response cascade were therefore ${S}_{HT,plateau}$, ${t}_{delayHTtodeath}$, and ${t}_{cellcyclearrest}$. Figure 1D shows the response cascade implemented to model the behaviour described.

Since cells are removed from the computational lattice independent of their position, this model allows reoxygenation and re-activation of previously quiescent cells. Due to the nature of the culture wells used (U-bottom shape), cells are, however, drawn towards the centre of mass resulting in a re-shuffling of the surrounding cells to fill any gaps. Figure 1C shows schematic images of heat-induced cell death mechanisms which resulted in a destabilization and disintegration of the spheroid for large thermal doses (few surviving cells), before the spheroid re-formed from the surviving cells.

### Implementation of radiation and hyperthermia combinations

**Figure 2:**
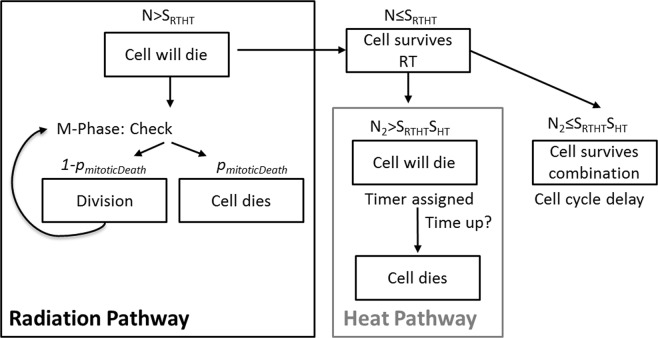
Response cascade used for modelling cell death dynamics in spheroids after combination treatments of radiation and hyperthermia. After treatment, the cell population is divided into surviving and dying cells by drawing random numbers $N$ and ${N}_{2}$ between 0 and 1 and comparing them to the clonogenic surviving fractions, ${S}_{RTHT}$ ($N$) and ${S}_{HT}$ (${N}_{2}$). Depending on the random number drawn, a cell will follow the route of radiation-induced cell death, heat-induced cell death, or survival.

Combination of RT and HT treatments were modelled by joining the two response mechanisms for single modality therapies described above. The combined clonogenic surviving fraction was ${S}_{RTHT}(d,{t}_{43})\cdot {S}_{HT}({t}_{43})$, where ${S}_{RTHT}(d,{t}_{43})$ was calculated using equation 2 with thermal dose dependent parameters $\alpha$ and $\beta$ as previously described^30^ to implement synergistic effects. ${S}_{HT}({t}_{43})$ is given by equation 4. The relevant response cascade is outlined in Fig. 2. A fraction of $1-{S}_{RTHT}$ follows radiation-induced cell death. Of the remaining cells, a fraction of $1-{S}_{HT}$ was simulated as undergoing heat-induced cell death. The remaining fraction, ${S}_{HT}\cdot {S}_{RTHT}$, survived, and continued proliferation after cell cycle delay.

### Parameter fitting and validation procedure

In the CA model, the diameter of the simulated spheroid was calculated as the average of the 100 maximum distances of cells to the spheroid centre in MATLAB (MathWorks, version 2017a). The coefficient of determination, ${R}^{2}$, was used as a measure of agreement between simulated and experimentally measured growth curves. Simulation parameters were adjusted to maximize ${R}^{2}$ in the calibration step using a grid search. Since the number of free parameters was limited as much as possible, each parameter controlled a specific aspect of the model and could thus be independently fitted to the specific data using a “one at a time approach”. Below, the specific features used for fitting each parameter are given in brackets. Since parameter cross correlations were not applied during the fitting procedure, multiple parameter combinations yielding a similar solution were not encountered in this approach. It should be noted that absolute parameter values refer entirely to an artificial, simplified biological system and thus should not be used for direct interpretation of biological meaning.

Cell growth was modelled with an allowance of 3 days for cell attachment and acclimatization relative to experimental growth curves. I.e. simulation started on day 3 in terms of the experimental time since seeding the cells and all growth curves are shown with the relevant time correction.

#### Oxygenation and untreated spheroid growth parameter estimation

Oxygen distributions within spheroids were compared qualitatively to histological sections stained with pimonidazole (for hypoxia), DAPI (for cell nuclei to show cell distribution) and Ki-67 (to show proliferation). For quantitative evaluation, simulations were compared with an analytical oxygenation model proposed by Grimes *et al*.^31^ for which parameters had previously been estimated for HCT116 spheroids^44,45^. This model provides a description of the partial oxygen pressure, ${p}_{O2}$, as a function of the radial distance to the spheroid centre, $r$, based on experimentally measurable dimensions within stained histological spheroid sections.

6 $${p}_{O2}(r)={p}_{0}\cdot \left(1+\frac{1}{{r}_{l}^{2}}\cdot \left({r}^{2}-{r}_{0}^{2}+2\cdot {r}_{n}^{3}\cdot \left(\frac{1}{r}-\frac{1}{{r}_{0}}\right)\right)\right)$$

Here, ${p}_{0}$ denotes the partial oxygen pressure in the culture medium (${p}_{0}\approx 100\,mmHg$^31^), ${r}_{l}$ is the diffusion limit, ${r}_{n}$ is the radius of the anoxic necrotic core, and ${r}_{0}$ is the outer spheroid radius. The diffusion limit ${r}_{l}$, i.e. the minimum radius of a spheroid with partial oxygen pressure equal to zero at its centre, can be measured experimentally since it is directly related to the width of the proliferating and quiescent zone, ${r}_{c}$, and the spheroid radius ${r}_{0}$.

7 $${r}_{l}=\sqrt{3{r}_{c}^{2}-\frac{2{r}_{c}^{3}}{{r}_{0}}}$$

According to the results presented by Grimes *et al*.^44^ HCT116 cells have ${r}_{l}=233\,\mu m$ and ${r}_{n}=155\,\mu m$. Using these values, the partial oxygen pressure as a function of the radial distance to the spheroid surface was calculated using equation 6. Results were compared with the simulated oxygen distribution using an oxygen consumption rate of 22.1 mmHg/s as proposed by Grimes *et al*., and the oxygen diffusion coefficient ${D}_{{O}_{2}}$ (gradient of hypoxia) was adapted to minimize the difference between central cross-sections of simulated and analytically calculated oxygen distributions.

Having calibrated the oxygen diffusion model, the parameters controlling untreated spheroid growth, i.e. initial number of cells (initial speroid diameter), cell doubling time (growth rate under full oxygenation), ${p}_{hypoxiaDeath}$ (onset of growth plateau), and ${p}_{clearNecrotic}$ (slope of growth plateau), were adjusted to maximise the agreement (in terms of coefficients of determination) between simulated and experimental growth curves of untreated HCT116 spheroids.

#### Treatment response parameter fitting and validation method

The experimental data for 10 Gy were used to determine the fitting parameters by adapting ${p}_{mitoticDeath}$, before and after an initial delay (slopes of the curve), ${t}_{delayRT}$ (peak location of the curve), to cell death after irradiation (free parameters: ${p}_{mitoticDeath < 0.5}$, ${p}_{mitoticDeath > 0.5}$, ${t}_{delayRT}$). The 10 Gy growth curve was chosen, since spheroids did not regrow after this treatment within the three week observation period. This simulation was not subject to uncertainties of surviving cells trapping dying G0 cells which simplified parameter fitting. After parameter fitting, growth curves after 2 Gy and 5 Gy irradiation were simulated without further parameter changes (validation). Similarly, free parameters ${S}_{HT,plateau}$ (time of regrowth), t_delayHTtoDeath_ (rate of shrinkage), and ${t}_{cellcycle}$ (onset of shrinkage), were fitted for HT treatment based on the 240 CEM${}_{43}$ growth curve and these parameters were then used to predict 40 and 80 CEM${}_{43}$ treatments. For combination treatments, no further changes to the simulation parameters were made and the growth curves after treatment with 2 Gy + 40 CEM${}_{43}$, 2 Gy + 80 CEM${}_{43}$, and 2 Gy + 160 CEM${}_{43}$ were simulated and compared to experimental data.

## Results

### Oxygen distribution and untreated spheroid growth

**Figure 3:**
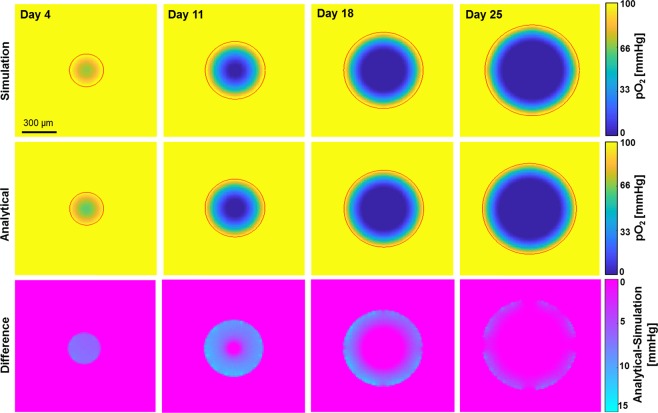
Comparison of iteratively simulated (top) oxygen partial pressure distributions within HCT116 spheroids of varying sizes with analytically calculated (second row) ones. Central planes are shown with red rings outlining the spheroid. The corresponding difference map (third row, analytical-simulation) revealed pressure differences of up to 15% between these distributions.

**Figure 4:**
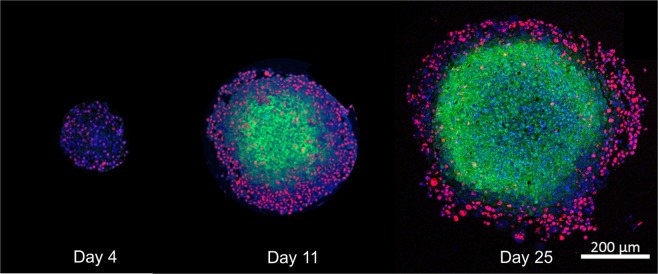
Histological sections of HCT116 spheroids fixed at three different time points after seeding, stained with pimonidazole (green, hypoxia), Ki-67 (red, proliferation), and DAPI (blue, all cell nuclei). No significant hypoxia is observed on treatment day (day four). A hypoxic core that is surrounded by a rim of cells many of which are proliferating (red) develops over time. Necrosis in the spheroid core may be indicated by a decreased cellular density at the centre in the section of a spheroid fixed on day 25.

 Figure 3 shows the simulated (fit parameter ${D}_{{O}_{2}}=3.8\cdot 1{0}^{-9}\,{m}^{2}{s}^{-1}$) and analytically calculated (model by Grimes *et al*.) equatorial oxygen distribution maps across the central cross section for spheroids of four sizes (284 $\mu m$, 500 $\mu m$, 664 $\mu m$, and 792 $\mu m$) corresponding to spheroids 4, 11, 18 and 25 days after seeding. Difference maps are shown in Fig. 3. Histological sections that showed qualitative agreement between hypoxic (i.e. pimonidazole staining) regions and simulated oxygenation levels are shown in Fig. 4. Simulated and analytically calculated distributions agreed with differences of less than 15%, the maximum deviations being observed in the outermost cell layers.

**Figure 5:**
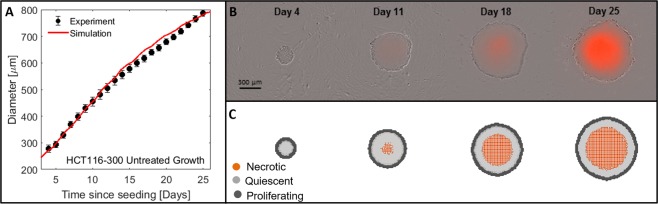
Experimentally measured growth curves of HCT116 spheroids and simulated curves using fitted parameters (**A**). Cell distributions in terms of live and dead cells were also compared between equatorial cross sections taken through simulated spheroids (**C**), and time-lapse microscopy images acquired on the Incucyte S3 (**B**) showing an overlay of phase contrast and PI fluorescence (orange). Simulated and experimentally measured images are shown on the same scale.

Model parameters controlling the formation of a necrotic core leading to a growth plateau were fitted with the best fit (${R}^{2}=0.99$) corresponding to ${p}_{hypoxiaDeath}=0.01$, ${p}_{clearNecrotic}=0.001$ and a doubling time of 28 h. The relevant experimental and simulated spheroid diameter growth curves are shown in Fig. 5 together with the simulated cell distributions and spheroid time-lapse microscopy showing an overlay of phase contrast and PI fluorescence (dead cells in orange) images. The spatial extent of dead cells at the core of the simulated and experimentally measured spheroid agree qualitatively.

#### Simulation of treatment response

**Figure 6:**
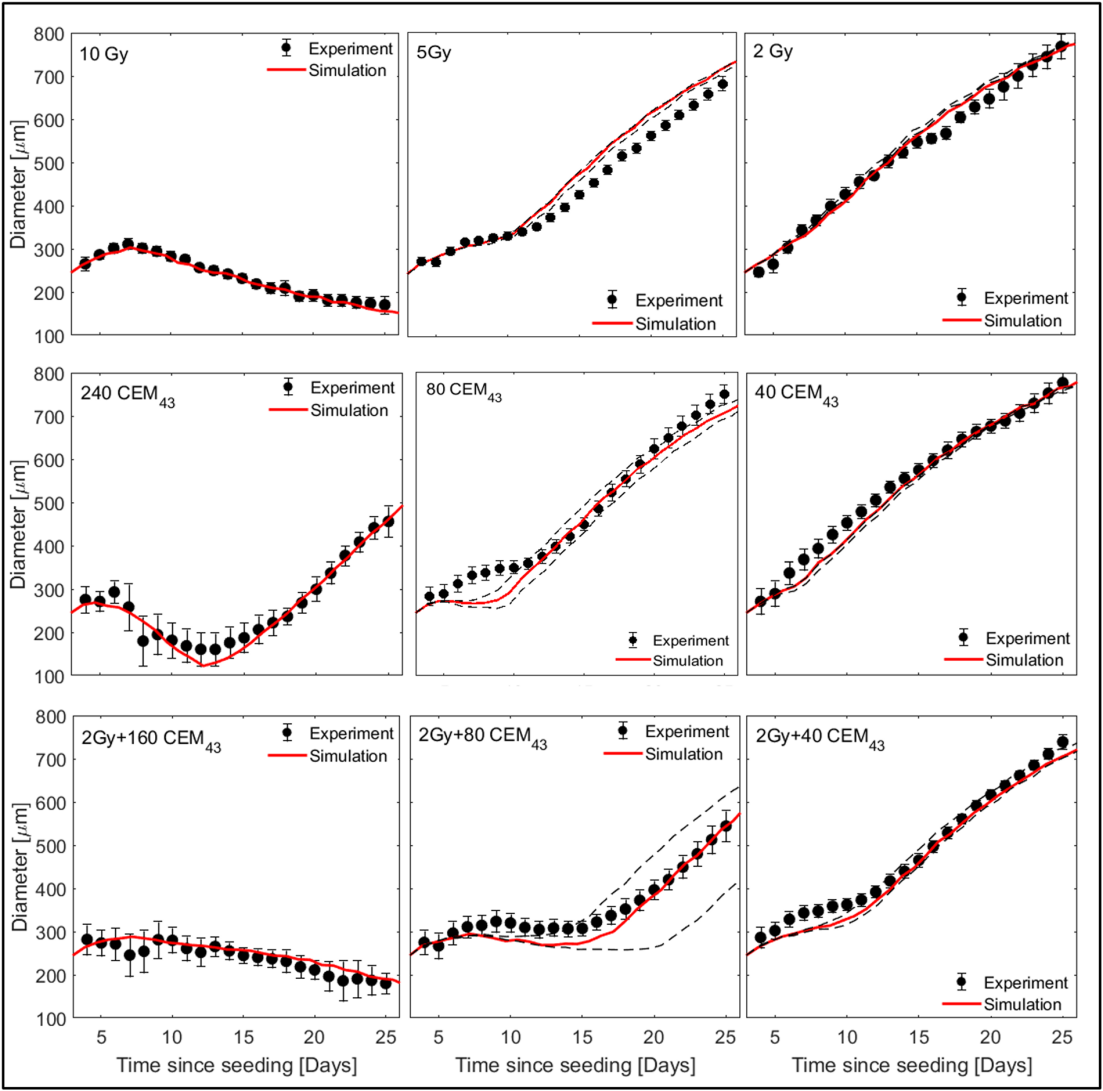
Comparison of simulated (lines) and experimentally measured (data points, mean values and standard errors of the mean of at least three repeat experiments) growth curves of HCT116 spheroids after irradiation (top row), hyperthermia treatment at 47${}^{\circ }C$ for 15, 5 and 2.5 min (middle row), and combination of 2 Gy radiation and hyperthermia at 47${}^{\circ }C$ for 10, 5 and 2.5 min (bottom row). Where applicable, dashed lines indicate simulation uncertainty ranges obtained by using upper and lower prediction bounds of the clonogenic surviving fraction. Simulation parameters were first adapted to match the growth curve after treatment with 10 Gy and 240 CEM${}_{43}$, i.e. calibration curves. Other simulations were performed without further modifications of the parameters used.

Parameter fitting to the 10 Gy growth curve was optimal (${R}_{10Gy}^{2}$ = 0.98) for an initial value of ${p}_{mitoticDeath < 0.5}=0.44$ during the first 3.5 days (i.e. ${t}_{delayRT}$) after treatment, and a value of ${p}_{mitoticDeath > 0.}=0.59$ thereafter. The results obtained, together with the validation data sets for 2 Gy and 5 Gy irradiated spheroids are shown in Fig. 6 (top). Results are shown for a representative simulation run using the mean surviving fraction (see Eq. 2). For 2 Gy and 5 Gy treatments, simulations were also performed using the mean $\pm$ one standard deviation of the clonogenic surviving fraction to show the variations in the data. Since 10 Gy was considered to be a treatment that killed all cells within the spheroid, no variation in the surviving fraction was accounted for. There was good agreement between simulated and experimentally measured data, for both the calibration and validation data sets (${R}_{5Gy}^{2}$ = 0.95, ${R}_{2Gy}^{2}$ = 0.98).

HT treatment parameters were fitted to be ${S}_{HT,plateau}$=0.0005, an average time delay to cell death, ${t}_{delayHTtodeath}$, of 96 h, and a mean cell cycle arrest duration of surviving cells of ${t}_{cellcyclearrest}=$30 h. Fig. 6 (middle) shows the comparison of simulated and experimentally measured growth curves after 240 (fit), 40 and 80 CEM${}_{43}$ treatments. A representative simulation run is shown with the uncertainty range of the clonogenic surviving fraction for 40 and 80 CEM${}_{43}$ treatments. There was a good agreement between simulated and experimental data (${R}_{240CEM43}^{2}$ = 0.95, ${R}_{80CEM43}^{2}$ = 0.94, ${R}_{40CEM43}^{2}$ = 0.98). The simulation under-predicted the size of heated spheroids (80 and 40 CEM${}_{43}$) within the first week after treatment. The observed lack of reduction in spheroid diameter may be due to either a loosening of the spheroid structure due to the majority of cells dying (${S}_{80CEM43}\approx 0.04$), or to a breakdown of the extracellular matrix which was not accounted for in the simulation. It was, however, considered that correct prediction of spheroid growth delay was most important, as was achieved (see Fig. 6 (middle)).

The simulation of combined RTHT treatments was carried out without further modifications of the parameters used. Simulated and experimentally measured spheroid growth curves are shown in Fig. 6 (bottom). There was good agreement for this data set (${R}_{2\,Gy+40\,CEM43}^{2}$ = 0.96, ${R}_{2\,Gy+80\,CEM43}^{2}$ = 0.88, ${R}_{2\,Gy+160\,CEM43}^{2}$ = 0.86).

## Discussion

Modelling spheroid response in terms of volumetric growth required close mimicking of the biological response observed experimentally. Despite using a deliberately simple implementation of a CA approach, it was possible to reproduce the most important features of the treatment of 3D tumour spheroids with radiation, hyperthermia, or both in combination. These included modelling oxygenation to predict the formation of a necrotic core and spheroid growth plateau, as well as implementing proliferation dependent radiation- and proliferation independent heat-induced cell death. It was shown that the simulation modelled experimental spheroid growth response to RT, HT and RTHT treatments (Fig. 6) (all ${R}^{2} > 0.85$) accurately.

Our framework represents an improvement over previous similar models^46,47^, both through the extent of experimental validation performed and the different treatment modalities simulated, i.e. RT, HT, and RTHT. Sophisticated biological interpretations^9,48^ and computational models^46,47^ have been proposed previously for the modelling of radiation response as a single modality treatment or in combination with chemo therapy. We identified and implemented the underlying cell death mechanisms between radiation and heating, and subsequently determined their influence on spheroid growth. We suggest that similar differences in cell death mechanisms are likely present for radiation and drug treatments due to different cellular targets, and it may be essential to consider dynamic cell death for such multi-modality therapies, to allow calculation of BEQD distributions for any kind of multimodality therapy.

One of the aims of the experimental calibration was to facilitate modelling of oxygenation effects. These include the formation of a necrotic core following prolonged hypoxia, the plateauing of spheroid growth, and the adaptation of the oxygen enhancement factors to capture the influence of hypoxia and normoxia in irradiated spheroids. The HCT116 spheroids used here were a good model for adapting the relevant response cascade as spheroids were well oxygenated on treatment day but developed a hypoxic and necrotic core over the observation period (three weeks). Although reference values were available for the oxygen consumption and diffusion rates within HCT116 spheroids^44^, using these values directly would imply that the voxel size used matched experimental cell sizes. Therefore, a small adaptation of the relevant diffusion rate was made, based on comparison with the analytical model proposed by Grimes *et al*. for modelling the partial pressure of oxygen. Despite this adaptations, differences of up to 15% in partial oxygen pressure were observed between the two approaches, with the analytical calculation providing higher values within the proliferating spheroid zone. Extreme values of 15% may be explained by a some individual cells lying outside the calculated mean diameter of the spheroid, whereas the analytical model assumes a perfect sphere. Although there was only a relatively homogeneous off-set between distributions on day four, larger spheroids showed only small differences with respect to the oxygenation gradients. These may originate from differences in the “growth history” of the spheroids used in the model by Grimes *et al*. and the ones used here. Grimes *et al*. used the liquid overlay technique for spheroid production and used spheroids 4 to 17 days after formation for their analysis and parameter estimation. This implies a larger range in incubation times, and a wider range of spheroid sizes obtained than the more standardised time points and spheroid formation method used here. There was qualitative agreement between simulated and experimental oxygenation levels estimated from histological sections, showing little significant hypoxia in small (day 4) spheroids, with increasingly hypoxic regions for larger spheroids (days 11 and 25). Parameters controlling the influence of hypoxia on treatment response, such as the OER, could not be fitted here, due to the absence of significant hypoxia on treatment day (see Fig. 4). Spheroids with hypoxic, but not necrotic, cores should be evaluated for this purpose. This could be achieved by treating the same HCT116 spheroid at a later time point after seeding.

Other than oxygen distribution, the current framework did not include modelling of other diffusive factors, such as glucose, waste products, or pH-levels that have been reported to affect heat sensitivity of monolayer cultures and cell viability in general and are therefore likely to affect treatment response of cells within a spheroid^49,50^. It should therefore be noted that the current formulation of “oxygenation” may indeed emcompass a number of diffusive processes to limit the number of model parameters. Accounting for separate description of oxygen and other diffusive factors could be the subject of future model developments if deemed necessary.

Another limitation of the frameworks was the type of simulation framework chosen, a cellular automaton model which intrinsically limited the ability to model some potentially relevant biological processes. Due to the fixed lattice size, it was not easy to implement treatment-induced variations in cell size, a loosening of the cellular structure (i.e. cell density variation), or mechanical compression of cells within the necrotic spheroid core. A potential solution to this problem would be the use of a lattice-gas model^29,51^, providing multiple cell occupancy for each constant sized voxel, independent of the number of cells present. This would allow modelling of varying cell density and simplify the simulation of cell migration that could not be considered here in addition to the constraint of a dense spherical structure. Despite this limitation, our model was able to describe the observed spheroid growth curves well, and we thus deemed it sufficient for the current purpose.

The ultimate goal for the simulation framework will be modelling of vascularized tumours *in vivo* and the simulation presented here represents a first step towards this. Using a systems oncology model based on the one presented here, calibrated for each hierarchically relevant environmental detail (i.e. cellular properties, inter-cellular interactions, 3D growth and microenvironmental influences) would allow comparison of the importance of intrinsic cellular properties, such as growth rate, cell cycle progression, and clonogenic treatment response, with the influence of microenvironmental factors such as hypoxia, pH, etc. This should help bridge the gap between experimental results observed *in vitro* in 2D or 3D cultures and those *in vivo*. It is generally agreed that hypoxic cells are more radioresistant than normoxic ones when evaluated by clonogenic assays. In an *in vivo* tumour, hypoxia would, however, coincide with elevated numbers of quiescent cells, increased cellular debris and local variations of interstitial pressure, all of which are known to impact RT treatment efficacy. It remains to be shown whether resolving only one of these problems, e.g. by increasing the overall level oxygenation, would actually enhance the response of the tumour as a whole. Systems oncology simulations performed for a well-characterized cell line can help to understand the importance of the individual mechanisms through sensitivity analysis better, and to provide a biologically weighted planning tool for the optimization of multimodality treatment scheduling and dosage. 3D tumour spheroids have been shown to recapitulate important features of tumours *in vivo*, such as gradients in oxygen and nutrients, or 3D cell-cell contact. However, a spheroid model such as the one presented here does not account for the impact of physiology, in particular tumour vasculature, surrounding normal tissue, and the clearance of cellular debris by the immune system. It is thus expected that the translation of this model to the description of tumour response *in vivo*, will be a complex and challenging undertaking. However, the spheroid model provides an important first step towards this goal

## Conclusions

Despite an implementation that deliberately simplified some aspects of biology, the simulation framework presented here was designed to take into account the most important differences between heat- and radiation-induced cell death in 3D tumour spheroids, namely the (in)dependence of cell death on proliferation. The framework was successfully calibrated and validated against experimental data from a commonly used human colon tumour cell line (HCT116). After further development and validation, the framework may provide the basis for more sophisticated simulation of tumour response *in vivo*, that could be used to optimise fractionation and scheduling of single or multi-modality treatments.

## Data Availability

The data sets used and/or analysed during the current study are available from the corresponding author on reasonable request. Once the project has been concluded in the group, the entire framework will be uploaded to a public code sharing platform.

## References

[1] Kampinga HH, Dikomey E (2001). Hyperthermic radiosensitization: mode of action and clinical relevance. International Journal of Radiation Biology.

[2] Pawlik TM, Keyomarsi K (2004). Role of cell cycle in mediating sensitivity to radiotherapy. International Journal of Radiation Oncology, Biology, Physics.

[3] Sugahara T (2008). Kadota Fund International Forum 2004. Application of thermal stress for the improvement of health, 15-18 June 2004, Awaji Yumebutai International Conference Center, Awaji Island, Hyogo, Japan. Final Report. International Journal of Hyperthermia.

[4] Horsman MR, Overgaard J (2007). Hyperthermia: a Potent Enhancer of Radiotherapy. Clinical Oncology.

[5] Dikomey E, Jung H (1991). Thermal radiosensitization in CHO cells by prior heating at 41–46C. International Journal of Radiation Biology.

[6] van Leeuwen CM (2018). Measurement and analysis of the impact of time-interval, temperature and radiation dose on tumour cell survival and its application in thermoradiotherapy plan evaluation. International Journal of Hyperthermia.

[7] Brüningk SC (2017). A comprehensive model for heat-induced radio-sensitisation. International Journal of Hyperthermia..

[8] Kok HP (2014). Quantifying the combined effect of radiation therapy and hyperthermia in terms of equivalent dose distributions. International Journal of Radiation Oncology Biology Physics.

[9] Kaida A, Miura M (2013). Visualizing the effect of tumor microenvironments on radiation-induced cell kinetics in multicellular spheroids consisting of HeLa cells. Biochemical and Biophysical Research Communications.

[10] Onozato Y, Kaida A, Harada H, Miura M (2017). Radiosensitivity of quiescent and proliferating cells grown as multicellular tumor spheroids. Cancer Science.

[11] Sutherland RM (1988). Cell and Environment Interactions in Tumor Microregions: The Multicell Spheroid Model. Science.

[12] Schwachöfer J (1990). Radiosensitivity of human melanoma spheroids influenced by growth rate. International Journal of Radiation Oncology Biology Physics.

[13] Durand RE, Sutherland RM (1972). Effects of intercellular contact on repair of radiation damage. Experimental Cell Research.

[14] Nath S, Devi GR (2016). Three-dimensional culture systems in cancer research: Focus on tumor spheroid model. Pharmacology and Therapeutics.

[15] Sankar PS (2017). Modeling nasopharyngeal carcinoma in three dimensions (Review). Oncology Letters.

[16] Duval K (2017). Modeling Physiological Events in 2D vs 3D Cell Culture. Physiology.

[17] Lauber K (2015). Targeting the heat shock response in combination with radiotherapy: Sensitizing cancer cells to irradiation-induced cell death and heating up their immunogenicity. Cancer Letters.

[18] Lauber K, Ernst A, Orth M, Herrmann M, Belka C (2012). Dying cell clearance and its impact on the outcome of tumor radiotherapy. Frontiers in Oncology.

[19] Wartenberg M (2005). Regulation of the multidrug resistance transporter P-glycoprotein in multicellular prostate tumor spheroids by hyperthermia and reactive oxygen species. International Journal of Cancer.

[20] Mahdavi SR, Yahyapour R, Nikoofar A (2016). Cytotoxic effects of hyperthermia, chemotherapy (Navelbine) and radiation on glioma spheroids. Radiation Physics and Chemistry.

[21] Asayesh T, Changizi V, Eyvazzadeh N (2016). Assessment of cytotoxic damage induced by irradiation combined with hyperthermia and Gemcitabine on cultured glioblastoma spheroid cells. Radiation Physics and Chemistry.

[22] Durand RE (1978). Effects of hyperthermia on the cycling, noncycling, and hypoxic cells of irradiated and unirradiated multicell spheroids. Radiation Research.

[23] Lücke-Huhle C, Dertinger H (1977). Kinetic Response of an In Vitro "Tumour-model"(V 79 spheroids) to 42 $${}^{\circ }$$ C Hyperthermia. European Journal of Cancer.

[24] Song AS, Najjar AM, Diller KR (2014). Thermally Induced Apoptosis, Necrosis, and Heat Shock Protein Expression in 3D Culture. Journal of Biomechanical Engineering.

[25] Richter K, Haslbeck M, Buchner J (2010). The Heat Shock Response: Life on the Verge of Death. Molecular Cell.

[26] Kampinga HH (2006). Cell biological effects of hyperthermia alone or combined with radiation or drugs: A short introduction to newcomers in the field. International Journal of Hyperthermia.

[27] Moreira J, Deutsch A (2002). Cellular Automaton Models Of Tumor Development: A Critical Review. Advances in Complex Systems (ACS).

[28] Ribba, B.T., Alarcon, T., Marron, K., Maini, P.K.& Agur, Z.*In: Sloot P.M.A., Chopard B., Hoekstra A.G. (eds) Cellular Automata. The Use of Hybrid Cellular Automaton Models for Improving Cancer Therapy*, vol. 3305 (Springer, Berlin, Heidelberg, 2004).

[29] Deutsch, A.Cellular Automaton Models for Collective Cell Behaviour.In 21th Workshop on Cellular Automata and Discrete Complex Systems (AUTOMATA).Lecture Notes in Computer Science, LNCS-9099, Cellular Automata and Discrete Complex Systems., 110, 10.1007/978-3-662-47221-72015

[30] Brüningk S (2018). Combining radiation with hyperthermia: a multiscale model informed by in vitro experiments. Journal of The Royal Society Interface.

[31] Grimes DR, Kelly C, Bloch K, Partridge M (2014). A method for estimating the oxygen consumption rate in multicellular tumour spheroids. Journal of The Royal Society Interface.

[32] Grimes DR, Partridge M (2015). A mechanistic investigation of the oxygen fixation hypothesis and oxygen enhancement ratio. Biomedical Physics and Engineering Express.

[33] Sendoel A, Hengartner MO (2014). Apoptotic cell death under hypoxia. Physiology.

[34] Mascheroni P (2016). Predicting the growth of glioblastoma multiforme spheroids using a multiphase porous media model. Biomechanics and Modeling in Mechanobiology.

[35] Delarue M (2014). Article Compressive Stress Inhibits Proliferation in Tumor Spheroids through a Volume Limitation. Biophysical Journal.

[36] Montel, F. *et al*.Isotropic stress reduces cell proliferation in tumor spheroidsNew Journal of Physics14055008201210.1088/1367-2630/14/5/055008 (2012).

[37] Freyer, J.P.Role of Necrosis in Regulating the Growth Saturation of Multicellular Spheroids. *Cancer Res*.**48**, 2432–2439, PublishedMay1988 (1988). 3356007

[38] Carreau A, Hafny-Rahbi BE, Matejuk A (2011). Grillon, C. andKieda, C. Why is the partial oxygen pressure of human tissues a crucial parameter? Small molecules and hypoxia. Journal of Cellular and Molecular Medicine.

[39] Fowler JF (1989). The linear-quadratic formula and progress in fractionated radiotherapy. British Journal of Radiology.

[40] Desouky O, Ding N, Zhou G (2015). Targeted and non-targeted effects of ionizing radiatio. Journal of Radiation Research and Applied Sciences.

[41] Gerweck LE, Gillette EL, Dewey WC (1974). Killing of Chinese hamster cells in vitro by heating under hypoxic or aerobic conditions. European Journal of Cancer.

[42] Gerweck, L. E., Nygaard, T. G.& Burlett, M.Response of cells to hyperthermia under acute and chronic hypoxic conditions. *Cancer Res*.**39**, 966âĂŞ972, PublishedMarch1979 (1979). 34477

[43] Blomsjö, M.et al.*Hyperthermia* (Blackie and Son Ltd., London, 1986).

[44] Grimes DR (2016). The role of oxygen in avascular tumor growth. PLoS ONE.

[45] Gomes A (2016). Oxygen partial pressure is a rate-limiting parameter for cell proliferation in 3D spheroids grown in physioxic culture condition. PLoS ONE.

[46] Zacharaki EI, Stamatakos GS, Nikita KS, Uzunoglu NK (2004). Simulating growth dynamics and radiation response of avascular tumour spheroids - Model validation in the case of an EMT6/Ro multicellular spheroid. Computer Methods and Programs in Biomedicine.

[47] Kempf, H., Hatzikirou, H., Bleicher, M.& Meyer-Hermann, M.{In Silico Analysis of Cell Cycle Synchronisation Effects in Radiotherapy of Tumour Spheroids. *PLoS Computational Biology*., **9**, 10.1371/journal.pcbi.10032952013 10.1371/journal.pcbi.1003295 PMC3828142 24244120

[48] Otani K (2016). Cell-cycle-controlled radiation therapy was effective for treating a murine malignant melanoma cell line in vitro and in vivo. Scientific Reports.

[49] Overgaard J (1976). Influence of extracellular ph on the viability and morphology of tumor cells exposed to hyperthermia. Journal of the National Cancer Institute.

[50] Freeman ML, Dewey WC (1977). Effect of pH on hyperthermic cell survival: Brief communication. Journal of the National Cancer Institute.

[51] Chopard B, Ouared R, Deutsch A, Hatzikirou H, Wolf-Gladrow D (2010). Lattice-Gas Cellular Automaton Models for Biology: From Fluids to Cells. Acta Biotheoretica.

